# The Technique of Gaining Entrance to Pulp Chambers and Root Canals

**Published:** 1900-08-15

**Authors:** L. P. Hall


DENTAL REGISTER.

Vol. LIV. AUGUST 15, 1900. No. 8.

### REPORT OF THE PROCEEDINGS OF THE FORTY-FOURTH ANNUAL MEETING OF THE MICHIGAN STATE DENTAL ASSOCIATION.

313-

### THE TECHNIQUE OF GAINING ENTRANCE TO PULP CHAMBERS AND ROOT CANALS.

BY L. P. HALL, D.D.S.

(*Continued from July No., page* 375.)

Place the rubber dam on three or four teeth in such a manner that the cavity will be about the middle of the teeth covered; then cut away all thin walls and excavate from cavity all debris. Next, with either burr or drill ream out, if necessary, that portion of dentin between canal and cervical border of cavity sufficiently to allow free access for probe or broach. Now cleanse the canal of all debris of whatever character, being careful not to forch either instruments or debris through apex of root. If canal is of usual type, no further reaming will be necessary; if much reduced in diameter, either a three or four-sided twist hand broach or a Gates-Glidden drill may be used. The twisted broaches as now made will cut out where the walls are not too hard, and the slender points will easily follow the curves usually found in these roots. Seldom is it well to force a drill to the extreme apex. Rather cut only enough to permit the free passage of broaches or probe with cotton for carrying medicinal agents or for drying purposes.

If, however, the operator expects to make use eventually of the canal for the post of a crown, it is advisable to so ream out the canal that it will admit a fair-sized platinum pin before filling, as it is more readily done while you have in mind the general shape and direction of canals than to trust to memory and follow only a slender point of gutta-percha or whatever may have been used as the filling material.

A tooth is occasionally presented in which there are either no cavities or the fillings are well made. In these cases it is of course best to open from the lingual side and in direct line with the long axis of the tooth. In case the tooth is very sore to touch, only the smallest and sharpest drill should be used; when it is once started, an even, steady pressure should be maintained. Once through to the pulp chamber, or as soon as this venting will permit, the opening should be enlarged so that not only will good free access be obtained to the canal, but also to the fan-shaped pulp chamber, that all debris may be removed from the crown portion of the tooth.

Superior cuspids may be entered in much the same manner. Usually they are entered from distal cavities, and if so the cavity should be extended well to the median line on the lingual side to avoid bending or binding of instruments. It must be remembered that these teeth have much longer roots, and, though usually straight, they may have much curve, even a right angle curve, near the apex. As they have large, strong roots, they will stand much reaming two-thirds of the way to the apex. Beyond that care must be exercised lest the drill be forced through the curve or to one side. A very fine spring-tempered probe may find the way through.

The first and second bicuspids are usnally entered through proximal cavities. Either mesial or distal cavi-ites, especially the distal, should be opened nearly to the center of the crown upon the long axis of the tooth, so a broach or drill will not be bent unnecessarily. The floor of the cavity should be opened sufficiently that no under-cut remains between the cavity and the pulp chamber.

In the first upper bicuspid there are almost invariably two canals if not two distinct roots. In the upper first bicuspid we have the pulp chamber more or less distinct from the root canals. These canals, situated at the extreme buccal and lingual sides of the pulp chamber, are usually quite slender and may be difficult to locate. A safe method is to use a rather large round bür and cut a clean base to the pulp cavity, carrying well to lingual and buccal, removing all sharp edges and overhanging shoulders of cavity opening. A minute smooth broach will usually locate these canals and give a fair estimate of curve, length and direction. The canals being located, they may now be opened with large Gates-Glidden drills, following with the next size until the canals are well opened. In the second bicuspid the pulp chamber and canal are less distinct, as the single canal is usually larger and extends from buccal to lingual of the chamber.

A bicuspid pulp sometimes dies after the tooth is well filled, or even when there is no cavity. In that case the tooth should be entered through the center of the crown with a small, sharp, plain drill, and this opening should be so enlarged that every portion of the pulp chamber may be cleaned. This opening is often made too small to allow of good access.

The buccal roots of the upper molars and the mesial roots of the lower first molars probably present the most difficulty.

A study of the anatomy of the teeth gives us some guide. In the first upper molars the roots are normally well apart, the anterior buccal root leaving the neck of the tooth as an extension of the mesio-buccal cusp or lobe, and curving to the mesial, while the disto-buccal root leaves the neck of the tooth as an extension of that lobe or cusp, but in a distal direction. The apices of these roots may turn toward each other more or less. The lingual root is larger and projects well into the palate.

In the second molars the buccal roots are closer together and leave the neck of the tooth on more parallel lines, though both may curve distally. The lingual root does not spread away so much to the lingual, and any two or possibly all three roots may unite.

In upper molars with fair sized mesial cavity there is no great difficulty of access, the main hindrance being in reaching the anterior buccal canal. Too often the posterior canal is mistaken for the anterior. If there is trouble in reaching this canal through the mesial cavity, how much more is there if we work through a distal cavity? Now surely we must open well into the crown of the tooth, even though we sacrifice a considerable portion of good tooth substance. All overhanging shoulders and undercuts must be cut out or beveled to orifice of main opening to enable operator to see every portion of floor of cavity, especially to openings of canals.

If there is no proximal cavity, gain entrance with a sharp, plain drill from the mesial side and toward central portion of crown, and enlarge this. If there is either mesial or distal cavity, open from this to central portion of crown. In either case, open.

In either upper or lower molars good access to pulp chamber may be had through large buccal cavities by extending into crown. If buccal margin is low, build up temporarily to hold dam. Having opened well the orifice through which to treat, excavate and cleanse. Now locate all the canals with a fine broach; in normal teeth the lingual is easily found, and so, perhaps, is the disto-buccal. The pulp chamber is usually located partly in the crown and partly in the neck of the tooth, so that the anterior buccal canal may be beyond neck of tooth, and in curve of root, hence far forward. If only one buccal canal is found, note its direction with regard to supposed direction of buccal roots, and then, with a large round bur in either a straight or right angle hand-piece, cut out in the direction the canal should be. Blow out chips and examine frequently with broach. Never use a small drill or small bur for locating a canal; there is too much danger of going in the wrong direction, and if a deep cut is made next to canal it troubles constantly when you pass into the right one. The orifice of the canal opening from the pulp chamber may be round or flattened, large or small. If small, and either round or flat, its orifice should be enlarged with largest Gates-Glidden drill so that instrumentation or treatment may be easily made. The rest of the canal may, if necessity demand, be enlarged with the next size drill until it can be cleansed and treated with a fair degree of ease and certainty.

Lower first molars are perhaps more trying than the seconds, as their roots divide closer to their crowns, and the mesial root, as it leaves the neck of the tooth, usually slants first to the mesial from pulp chamber, thus bringing the openings of the canals well under the shoulder of dentin between the front of the tooth and the pulp chamber. Hence enough tooth substance should be cut away to give the operator a chance to reach that canal from a distil direction. Too much emphasis can not be laid on the question of proper extension of cavity for access. In lower second molars the canals are closer together and more nearly parallel.

The canal portion of the lower incisor is much flattened from labial to lingual; the longest diameter is at the gingeval line. It grows thinner as it nears the cutting edge, but somewhat broader from mesial to distal. It contracts gradually toward the root and blends with the canal in a slit-like opening extending the greater portion of the root. In many instances, however, on account of the thinness of the root, the canal may be divided for a portion, or possibly for all the way. This division may begin at the gingeval line or below it, usually uniting into a single apical foramen. As age advances these canals often become exceedingly minute. Access may be gained through a mesial or distal cavity, which must be extended pretty well toward the cutting edge, or through the lingual side, in line of the long axis of the canal. Here again must the shoulder, formed between the canal opening and the cervical border, be cut away so an instrument may pass with little or no bending. On account of the thinness of the root from mesial to distal, great care must be exercised that a drill is not forced through the lateral wall. Cavities must be extended toward the cutting edge enough to permit thorough excavation of the pulp chamber.

Lower cuspids, when treated, are entered in a similar manner, i.e., either through a proximal cavity or through the center of the lingual side. The same care must be given to cutting away the shoulder or borders to allow of good, free access in line of long axis of canal. In the lower cuspid it is well to bear in mind that there may be two distinct canals with separate apical foramina. Should the entrance be from a proximal cavity that did not open much on the labial side or cutting edge, it should be extended far enough to lingual to give most direct access to canal.

Lower bicuspids are often difficult of access because of their position in the jaw, i.e., the position of crown on root, and because of the fact that their buccal cusps are carried so far over to lingual, thus bringing the thickest portion of enamel over the central or long axis of the canal. In extending either a mesial or distal cavity toward the center of the crown, it can be done by cutting up from beneath, that is, by undermining the dentin and then cleaving off the enamel. When a lower bicuspid must be entered either through the crown or through a good filling, extreme caution will be necessary lest the drill is forced through the lingual wall at neck of the tooth, as the crown is placed on the root at a marked angle, and the neck of the tooth is much constricted.

Access is not unfrequently obstructed by pulp stones. In the single-rooted teeth these deposits are usually round and easily dislodged. In molar teeth they may be small and round, or they may be large and irregular, nearly filling the pulp chamber. In the latter cases, with a good general knowledge of the size and shape of the pulp chambers, there can be no harm in cutting away freely with large, round bur until the edges of this deposit or growth can be outlined. Then it can be pried out or chipped away.

Sometimes a case is presented in which there is exposure of pulp through a small, deep cavity, say on the buccal side of a tooth, close to cervical margin—a cavity that can be excavated and filled, but through which it would be impracticable to remove a pulp or treat a tooth. In such cases excavate and apply treatment for devitalization of pulp, and fill permanently with expectation of opening through a more convenient point of access, when the pulp chamber may be extended to original treatment, which may be now removed; the chamber may now be extended to a good, sound portion of filling without cutting away the coronal portion over original cavity.

The position of the patient in the chair is of great importance in treatments. For upper treatments the patient should sit well back in chair, with head thrown back. The chair may now be tilted back so the operator may have a good view of work.

In lower tooth treatments the patient should sit well back in chair, with head nearly erect, so that no shadow is cast by the lower lip or the lower front teeth.

As an aid in finding canals and gaining access, dilute sulphuric acid, followed by bicarbonate of soda, is often used. The action of the acid is not only to disintegrate the decomposed pulp structure, but to enlarge the canals by the solvent action of the acid on lime tissues. The acid is neutralized by the bicarbonate of soda, the effervescence so caused bringing out many small particles of debris not otherwise reached by broaches. Again, when neutralization and effervescence have ceased and the cavity has been dried by hot blast, the walls of cavity and canals are covered with a thin deposit of soda, and a canal will sometimes show a dark spot which has not been noticeable before.

In the writer's opinion it is not advisable to use sulphuric acid in recently devitalized teeth; 3 percent pyrozone, or peroxide of hydrogen, or peroxide of sodium, may be used.

DISCUSSION.

Discussion on Dr. Hall's paper opened by DR. A. C. RUNYAN, South Haven, Mich.

*Mr. President, Ladies and Gentlemen:*—I wish to say that I feel highly honored in being called upon to open the discussion on Dr. Hall's paper, but the Doctor has covered the ground so thoroughly and has shown such thorough knowledge of his subject, both in the anatomy of the teeth and technique of opening into the pulp chambers and nerve canals, and has the faculty of presenting it in such thorough and lucid terms in his paper that there is very little if anything for me to say. I certainly have no criticisms to make, and I believe if we follow the directions he has so carefully laid down there will be much less trouble in treating abscessed teeth.

There are a few points that I have thought might be well to emphasize by recalling them to your attention.

First. Those cases where the cavity extends to the cervical margin and under the gum, making it impossible to adjust the rubber dam. His method of filling such cavities at once and making a new opening through the crown for treatment is a very good suggestion, and makes it possible to approach aseptic treatment where otherwise it would be difficult.

In regard to the Gates-Glidden drills, personally I have never felt that I had acquired sufficient manipulative ability to use them and be safe. However, I believe that in some hands they may be used to advantage I avoid using them by flushing the cavities with dilute sulphuric acid and neutralizing with bicarbonate of soda, as the essayist suggests, which will usually locate the openings of the canals; then, with the finest Donaldson broach I carefully open it, applying more acid from time to time by dipping the broach into the acid and carrying it into the tooth.

Those of you who have not tried it will be surprised to find how easily you can gain access to apparently impenetrable canals. By the use of sulphuric acid you are accomplishing several things at once. It is a thorough disinfectant and a good styptic and a bleacher.

The Doctor's manner of applying the rubber dam on more than one or two teeth is a good suggestion, as we can have much more room to work and get a better lighting into the cavities. In fact, there is no point made in the paper or suggestion offered, which if thoroughly followed, but will give us good results and make our patients feel that they are in the hands of professionals.

Dr. Wood: I think the essayist stated that in anterior teeth where he wished to insert a Logan crown or a crown with any pin previous to the filling of the root, he would ream out the canal. Now, I have tried that in my short experience and have abandoned it for the method I now use of first filling that canal before preparing it for the pin of the crown I do not believe in enlarging it at all, I believe that the smooth sides of the canal will allow the passage of the pin better than any large opening I may make. In fact, in reaming out the canal first it is impossible to get the filling material to the apex of root, it fills up, when you force the point up it does not fill to apex of root; that is an important point, and I have not been able to do it as he says

Dr. Crouse: May I ask a question. Do I understand Dr. Hall to say he treats all pulp canals the same, whether the pulps have been recently taken out or in canals that have decayed?

Dr. Hall: I treat them all this way when it is necessary to get free access, it does not matter whether it is a recently devitalized or suppurating pulp.

Dr. Crouse: I consider it bad practice to ream out canals, and I also consider it bad practice to use the sulphuric acid as you do

Dr. Hall: I do not consider it advisable to use the sulphuric acid in all cases.

Dr. Oakman: When I was at college I was taught never to put a drill into a canal in trying to remove the pulp, but since graduating, particularly the last four years, I have become addicted to the use of the Gates-Glidden drill, and have found nothing so useful. Treating of root canals was my bug-bear. The use of the drill, as Dr. Hall has put it, I think is the correct way. It is valuable for getting pulp out, and also for getting the canal ready to fill. By using the large drills first and working on down to the smallest the best results are assured.

Dr. Crouse: May I tell you how to take a pulp out? I think it is bad practice to drill out and disturb the natural surface of the root canal. Take two or three shreds of cotton, wind around broach and thrust it into the canal and take pulp out. I can do it forty-nine times out of fifty. I would not give a cent for a Gates-Glidden drill. I place the cotton on broach, the nerve or pulp will adhere to the cotton.

Dr. Hall: What do you do when the canal is constricted and you can not possibly get a broach into it? You also object to sulphuric acid, which was suggested in the paper for getting access to the cavity. I would ask, why not use sulphuric acid?

Dr. Crouse: If the acid stops before it gets to the end of the root it is all right, but if allowed to go further it is extremely uncomfortable.

Dr. Hoff: I think we make a mistake in using the drills when we do not know how to use them. Dr. Hall tells us to use the large drill first and then the smaller ones. You read the advertisement in Dr. Crouse's journal which tells you to use the small one first then the larger and so on, which is all wrong. If you use them correctly, as Dr. Hall suggests, they will not break. There are roots into which it is impossible to get broaches without reaming them first. A Gates-Gliden drill revolved fast in the engine will cut the hard, overhanging edges out so that you can then get a broach in clear. If you insert a broach wound with cotton in such cases the pulp will not come out entire but will be torn, and it will be impossible to take it all out without enlarging the canal. The drill, properly used for reaming pulp canals, is not objectionable but almost invaluable. I broke one of them off in a canal once but had no difficulty in removing it. I use them all the time and could not do without them.

Dr. Corns: It is possible we do not understand Dr. Hall's idea. I do not think that he meant to say that he drills into the straight root canals where he has a large opening, but that he uses them only for cases where the canal is twisted or small.

Dr. Hall: I sometimes remove the greater portion of the pulp with the Gates-Glidden drill. I can go up into the canal of an incisor or molar and take out nearly all the contents of the pulp canal with the drill. I then use the broach to take out any which may remain.

Dr. Diack: My childhood days were regaled with the stories from "Arabian Nights," and I used to wonder at the remarkable deeds in those stories and as I grew older I often think of those tales. And so, when I first heard how the older operators sneak up along the side of that little inoffensive nerve with a broach and take it out whole, I said my days of fairy tales have returned again. I used to meet together with my brother dentists to compare experiences, and we always had to condole with one another about not being able to get out those little bits of pulp in the bottom of root canals. And so, when I again hear to-day how it is possible to sneak up the side of the pulp and take it out whole, I am quite certain my "Arabian Nights" have returned.

Dr. Root: There are all sorts, forms and conditions of drills and broaches, and I want to be honest and say that in Grand Rapids there are some of these broaches in the roots of teeth, and I did not put them all there either I want to make this statement, and I do it without any fear of successful contradiction, that there is a large percentage of the buccal roots of upper molar teeth that are never completely filled.

Dr. Hall: I did not say I used these drills everywhere, but rather where they can be used to advantage, and in such cases I do not hesitate to use them. I have broken some drills, but before using them I broke all kinds of broaches. I do not break so many now as I used to. We all make mistakes, and we make some in treating the root canals, but I am confident we get enough good out of these drills to warrant their use. If it is necessary in the anterior teeth I ream out the canals, but not in every case, as some here think. I asked one who was condemning the use of these drills how he used them. He said, I use the small one first, and then the next one in size, etc. I told him if he would reverse that he would keep on using them I have seen other people who made things and did not know how to use them. There is a manufacturer who makes clamps but he does not know all about them.

In regard to removing the pulp from the canal intact every time reminds me of a gentleman whom I have often heard tell how he takes a fine broach and turns a delicate little hook on the end and then slides it up alongside of the pulp and gives a dextrous little twist and pulls out the pulp whole. Now I have never been able to do that, there is nearly always a part of the pulp remaining after the attempt and it is necessary to ream out the canal to remove it.

I do not drill out the nerve and pulp as somebody else has said he did. I take it out intact if possible. I do not want it ground up into a mass, I want it whole and clean and if it comes out intact I am satisfied with the canal, because in that case I know I can make my treatments and fill the canal with gutta-percha also. But if I can not get it out, I want to drill the canal for access so that I may remove the remains. I have a fine broach which I use in determining the direction of the canals.

Question: Do you use that before you remove the pulp?

Dr. Hall: No, I get the pulp out if I can, and if I do not I introduce this fine broach and determine the direction and length of the canal. The broach is spring-tempered and follows the deviations of the canal, and when it comes out it will retain a sufficient amount of that curvature to give me something of an idea the way it went in. I use the Ivory twist broach made like a shaving when I can. If it is a canal which I can easily enter I twist this broach into the pulp and draw it out. These break occasionally, but I think they are good things to bring out the pulp and if carefully used are very effective.

Dr. Crouse: I am not a Christian Scientist, but I believe in my patients having it. This is a true story and it is not made up out of whole cloth. I had a lady patient who had had a number of children. She always went home from my office to suffer for three or four days with shock from any dental operation, no matter how mild. She finally got hold of Christian Science, and the first time afterward she took her place in my chair and said, "I do not want you to ask me if it hurts, nothing hurts any more." It is a fact that she now goes home without any prostration whatever. I wanted to put a bridge on a lower bicuspid and molar, and I had to grind down the bicuspid. I started grinding, watching the patient all the while, and she did not make a start; you all know one of those corundum wheels is not a very pleasant thing used in this way. By and by after grinding quite a while I saw a little hole. I took a very small broach and pulled the pulp out alive as clean as a whistle. And so, I say the dentist who does not advocate Christian Science to his patients does not know what he is talking about.

---

Third session called to order at 9:15. After reading of minutes of last meeting, Dr. Higgins, of Traverse City, rea l a paper on "Pressure Anesthesia."

